# The Diagnostic Value of Serum PIVKA-II Alone or in Combination with AFP in Chinese Hepatocellular Carcinoma Patients

**DOI:** 10.1155/2021/8868370

**Published:** 2021-02-08

**Authors:** Fei Xu, Lulu Zhang, Wenting He, Di Song, Xiaomeng Ji, Jianyong Shao

**Affiliations:** ^1^State Key Laboratory of Oncology in South China, Collaborative Innovation Center for Cancer Medicine, Sun Yat-sen University Cancer Center, Guangzhou 510060, China; ^2^Department of Molecular Diagnostics, Sun Yat-sen University Cancer Center, Guangzhou, China

## Abstract

**Background:**

At present, the diagnostic accuracy of alpha-fetoprotein (AFP) for hepatocellular carcinoma (HCC) surveillance is insufficient. It remains controversial whether prothrombin induced by vitamin K absence II (PIVKA-II) has a better diagnostic value than AFP for HCC patients.

**Objective:**

To investigate the diagnostic role of PIVKA-II alone or in combination with AFP in Chinese HCC patients.

**Methods:**

Serum AFP and PIVKA-II levels were detected and analyzed in 308 HCC afflicted patients and 120 unafflicted controls. The receiver operator curve (ROC) and area under the curve (AUC) were conducted to evaluate the clinical value of AFP and PIVKA-II for diagnosing HCC and early HCC.

**Results:**

In the whole HCC cohort, the diagnostic values of PIVKA-II were better than that of AFP. The AUC of PIVKA-II and AFP was 0.90 (95% CI 0.87-0.94) and 0.79 (95% CI 0.74-0.84), respectively. “AFP + PIVKA-II” yielded a high sensitivity of 95.1% and a specificity of 83.3%, with the AUC 0.89 (95% CI 0.85-0.93). In the early stage HCC group, the diagnostic accuracy of PIVKA-II was also better than that of AFP. The AUC of PIVKA-II and AFP was 0.83 (95% CI 0.77-0.89) and 0.75 (95% CI 0.68-0.81), respectively. “AFP + PIVKA-II” achieved the sensitivity of 83.3% and specificity of 89.1%, with an AUC of 0.86 (95% CI 0.81-0.91). Moreover, for AFP-negative HCC patients, serum PIVKA-II showed good diagnostic performance, with an AUC of 0.804 (95% CI 0.720-0.887). Besides, elevated PIVKA-II level was a strong independent risk factor for HCC patients with portal vein tumor thrombus (PVTT) (OR = 4.890, *P* = 0.020).

**Conclusion:**

PIVKA-II is superior to AFP in HCC screening, and AFP in combination with PIVKA-II significantly improves the diagnostic value for Chinese HCC patients. PIVKA-II could effectively indicate HCC accompanied by PVTT and help to optimize the therapeutic strategy.

## 1. Introduction

Hepatocellular carcinoma (HCC) is the sixth frequently diagnosed malignancy and the third leading cause of cancer-related death worldwide. Although the management of HCC has improved in recent years, the long-term overall survival remains poor [1–3]. Thus, sufficient methods are urgently needed for screening HCC at an earlier stage. Among traditional diagnostic and prognostic biomarkers, serum alpha-fetoprotein (AFP) level is the most frequently used for HCC detection and surveillance. However, the diagnostic accuracy of AFP for HCC patients is unsatisfactory. Previous results showed that serum AFP yielded 39-65% sensitivity and 76-94% specificity in detecting HCC, which is still far from ideal for clinical application [4, 5].

Prothrombin induced by vitamin K absence II (PIVKA-II) is firstly described in 1984 as a biomarker specific for HCC [6]. Increasing evidence has indicated that PIVKA-II demonstrated good diagnostic and prognostic values for HCC surveillance [7–9]. However, there are also some studies which supported that AFP performs better than PIVKA-II for HCC screening [10, 11]. Thus, results regarding the diagnostic efficiency of PIVKA-II in comparison or in combination with AFP still remain controversial. Currently, serum PIVKA-II level is considered a promising diagnostic biomarker in Japanese HCC patients. However, the clinical applications of serum PIVKA-II to assess Chinese HCC patients are limited. Thus, more studies are necessary to provide more evidence investigating the diagnostic accuracy of PIVKA-II in Chinese HCC patients.

Portal vein tumor thrombus (PVTT) is the most common macrovascular invasion and a frequent complication of HCC. Previous studies reported that the incidence of PVTT in HCC patients was 44%-62.2%. HCC complicated by PVTT is associated with limited treatment approaches, increased risk of recurrence, and poor prognosis [12–14]. Thus, predicting portal vein tumor thrombus is crucial for making cure strategies and prognosis assessment. Accumulating evidence suggested that PIVKA-II could stimulate overexpression of vascular endothelial growth factor (VEGF) and related to PVTT occurrence [15, 16]. However, lately, few studies explored the predictive role of PIVKA-II for HCC accompanied by PVTT.

Therefore, this present study is aimed at investigating the diagnostic role of serum PIVKA-II alone or in combination with AFP for Chinese HCC patients, and to determine the relationship between serum PIVKA-II level and PVTT.

## 2. Methods

### 2.1. Study Settings and Patients

**Figure 1:**
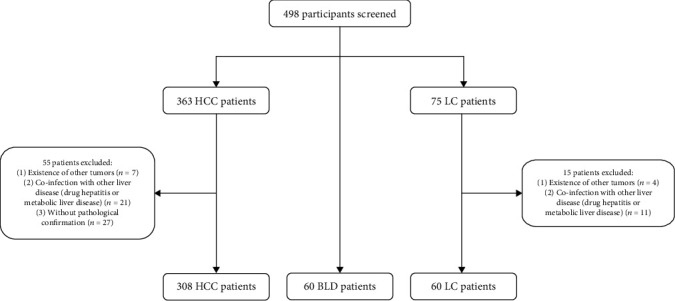
Flowchart depicting the selection process of the participants.

The present retrospective study enrolled a total of 428 subjects, including 308 HCC patients, 60 patients with HBV-related liver cirrhosis (LC), and 60 patients with benign liver disease (BLD). HCC was defined according to the American Association for the study of Liver Disease (AASLD) Practice Guidelines for the management of hepatocellular carcinoma (updated version, 2010). BLD cases were patients with hepatic cysts or hepatic hemangioma confirmed by ultrasound or computed tomography. The BCLC (Barcelona Clinic Liver Cancer) staging system was used to assess the stage of HCC patients, and early HCC was defined as BCLC 0-A stage. Sun Yat-sen University Cancer Center approved this retrospective, anonymous analysis of data, and the requirement for written informed consent was waived. The flowchart depicting the selection process of the enrolled participants is shown in Figure 1.

### 2.2. Measurements of AFP and PIVKA-II

Serum AFP levels were detected by the electrochemiluminescence immunoassay according to the manufacturer's manual (Roche Diagnostics, GmbH, Mannheim, Germany). Serum PIVKA-II levels were detected by the chemiluminescence enzyme immunoassay according to manufacturer's manual (Fujirebio, Inc., Tokyo, Japan). The cut-off values for serum AFP and PIVKA-II levels were 25 ng/ml and 40 mAU/ml, respectively.

### 2.3. Statistical Analysis

All statistical analyses were performed using SPSS version 22 software (IBM, Armonk, USA). The Mann-Whitney test, Kruskal-Wallis test, chi-squared test, and Fisher's exact test were used, as appropriate, to facilitate comparisons of differences in data between groups. Receiver operating characteristic (ROC) curves and area under the curve (AUC) with 95% CIs were conducted to elucidate the diagnostic values of AFP and PIVKA-II. Diagnostic efficiency-related parameters including sensitivity, specificity, positive predictive value (PPV), negative predictive value (NPV), Kappa value, and accuracy were calculated. Logistic regression analyses were used to facilitate investigations of the relationship between PIVKA-II levels and recorded clinical variables of HCC patients. *P* < 0.05 was regarded as statistically significant.

## 3. Results

### 3.1. Patient Characteristics

A total of 428 participants including HCC patients (*n* = 308), LC patients (*n* = 60), and BLD cases (*n* = 60) were recruited from June to September 2019. Among 308 HCC patients, 276 were HBV-related HCC. The median PIVKA-II and AFP levels were significantly higher in HCC patients compared with LC and BLD patients (*P* < 0.05). The clinicopathologic parameters of all participants are summarized in Table 1.

### 3.2. Diagnostic Performance of AFP and PIVKA-II in HCC Patients

**Figure 2:**
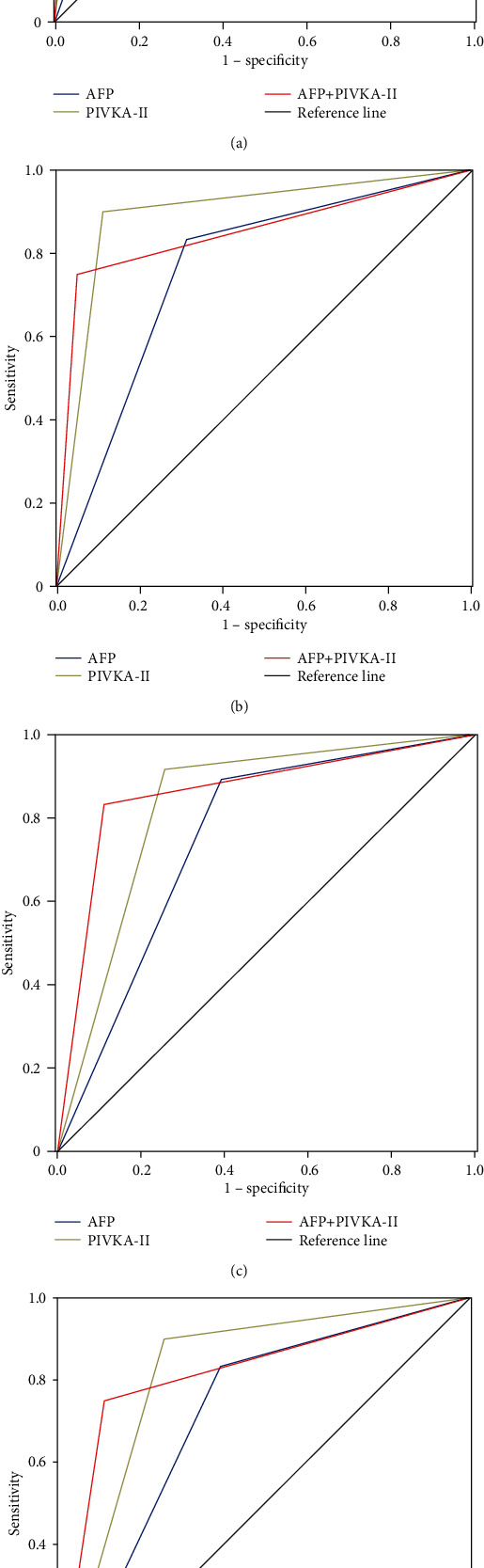
Diagnostic values of AFP and PIVKA-II for HCC. (a) ROC curves of AFP, PIVKA-II, and AFP + PIVKA-II for diagnosis of whole HCC cohort versus all controls; (b) ROC curves of AFP, PIVKA-II, and AFP + PIVKA-II for diagnosis of whole HCC cohort versus HBV-related liver cirrhosis patients; (c) ROC curves of AFP, PIVKA-II, and AFP + PIVKA-II for diagnosis of early stage HCC versus all controls; (d) ROC curves of AFP, PIVKA-II, and AFP + PIVKA-II for diagnosis of early stage HCC versus HBV-related liver cirrhosis patients.

To investigate the diagnostic accuracy of AFP and PIVKA-II in overall HCC patients, LC and BLD cases as cancer-free controls were pooled together for analysis. Our results indicated that PIVKA-II presented a better performance than AFP for diagnosis of HCC. The sensitivity, specificity, PPV, NPV, Kappa, and AUC of PIVKA-II were 89.0%, 91.7%, 96.5%, 76.4%, 0.76, and 0.90 (95% CI 0.87-0.94), respectively. For AFP, the sensitivity, specificity, PPV, NPV, Kappa, and AUC were 68.8%, 87.6%, 94.2%, 52.7%, 0.48, and 0.79 (95% CI 0.74-0.84), respectively. When AFP and PIVKA-II were used in combination, the results yielded a high sensitivity of 95.1% and specificity of 83.3%, with the AUC 0.89 (95% CI 0.85-0.93). When trying to discriminate HCC from LC patients, PIVKA-II also showed a better diagnostic role than AFP. The sensitivity, specificity, and AUC of PIVKA-II were 89.0%, 90.0%, and 0.89 (0.85-0.94), respectively. For AFP, the sensitivity, specificity, and AUC were 68.8%, 83.8%, and 0.76 (0.70-0.82), respectively (Table 2). ROC curves were illustrated as shown in Figures 2(a) and 2(b).

### 3.3. Diagnostic Performance of AFP and PIVKA-II in Early Stage HCC Patients

Diagnostic values of PIVKA-II or “AFP + PIVKA-II” were also better than that of AFP alone for early HCC patients. For AFP, the sensitivity was 60.9%, specificity was 89.2%, Kappa value was 0.50, and AUC was 0.75 (95% CI 0.68-0.81). For PIVKA-II, the sensitivity was 74.5%, specificity was 91.7%, Kappa value was 0.67, and AUC was 0.83 (95% CI 0.77-0.89). Moreover, the combined “AFP + PIVKA-II” biomarker achieved dramatic diagnostic efficiency, with a sensitivity of 83.3%, specificity of 89.1%, AUC of 0.86 (95% CI 0.81-0.91), and Kappa of 0.72. When trying to discriminate HCC from LC patients, PIVKA-II also exhibited better diagnostic performance than AFP. The sensitivity, specificity, and AUC of PIVKA-II were 74.5%, 90.0%, and 0.82 (0.75-0.88), respectively. For AFP, the sensitivity, specificity, and AUC were 60.9%, 83.3%, and 0.72 (0.64-0.80), respectively (Table 3). ROC curves were illustrated as shown in Figures 2(c) and 2(d).

### 3.4. Diagnostic Performance of PIVKA-II in AFP-Negative HCC Patients

**Figure 3:**
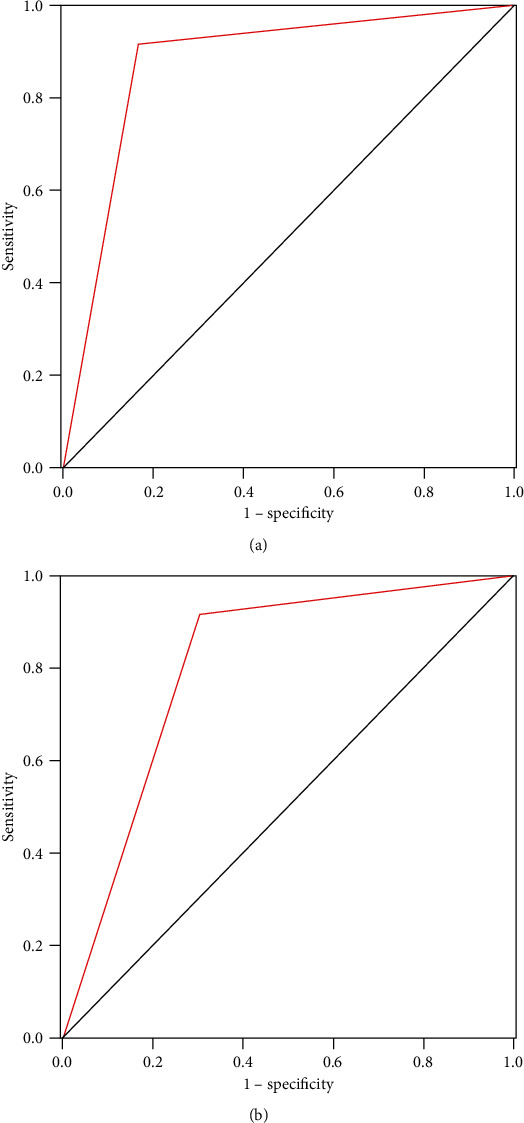
Diagnostic value of PIVKA-II for AFP-negative HCC patients. ROC curves of PIVKA-II for diagnosis of HCC (a) and early stage HCC (b) in AFP-negative HCC patients; the AUC were 0.88 and 0.81, respectively.

It was worthy of note that PIVKA-II had a good diagnostic performance for AFP-negative patients diagnosed with HCC. The AUC for overall HCC diagnosis was 0.88 (95% CI 0.823-0.927), with a sensitivity of 83.3%. The AUC for early HCC diagnosis was 0.81 (95% CI 0.72-0.89), with a sensitivity of 69.8%. Our study revealed that PIVKA-II was an excellent diagnostic biomarker even in the AFP-negative HCC group. ROC curves were illustrated as shown in Figure 3.

### 3.5. Correlation between PIVKA-II and HCC Clinicopathologic Parameters

To explore the relationship between serum PIKVA-II level and clinical parameters of HCC, all HCC patients were divided into the low PIVKA-II expression group (*n* = 30) and the high PIVKA-II expression group (*n* = 278) according to the cut-off value of 40 mAU/ml. Our study showed that serum PIVKA-II level was significantly correlated with tumor size (*P* < 0.001), tumor number (*P* = 0.01), lymphatic metastasis (*P* < 0.001), BCLC stage (*P* < 0.001), tumor differentiation (*P* = 0.027), and portal vein thrombosis (*P* = 0.01). Besides, we found that HCC patients with age ≤ 50 years were more likely to have higher serum PIVKA-II levels (*P* = 0.002). However, the differences of PIVKA-II serum levels in the distant metastasis group and the microvascular invasion group were not significant (Table 4). Further univariate and multivariate analyses indicated that the elevated PIVKA-II level was independently associated with an increased risk of greater tumor size (OR = 4.280, 95% CI 1.952-9.384, *P* = 0.012) and portal vein tumor thrombus (OR = 4.890, 95% CI 1.361-38.169, *P* = 0.020) (Table 5).

### 3.6. Predictive Factors for PVTT

Among all 308 HCC patients, 65 patients have portal vein tumor thrombus. As summarized in Table 6, our results revealed that the age (OR = 0.414, 95% CI 0.214-0.799; *P* = 0.009), tumor multiplicity (OR = 2.118, 95% CI 1.097-4.089; *P* = 0.025), and PIVKA-II > 40 mAU/ml (OR = 4.890, 95% CI 1.361-38.169; *P* = 0.020) were statistically associated with PVTT. It is worthy of note that PIVKA-II as the only serum biomarker could independently predict the increased risk of PVTT.

## 4. Discussion

Currently, AFP is the most clinically applicable biomarker for HCC detection. However, its unsatisfactory diagnostic efficiency is far from meeting the clinical requirement. Our research is aimed at identifying and validating effective noninvasive biomarkers for HCC management, and our previously published study has indicated that hypermethylation of SCAND3 and Myo1g gene was a potential diagnostic biomarker for hepatocellular carcinoma [17]. Recently, PIVKA-II is emerging as a promising marker for diagnosing HCC, but it is not as the routine analysis for HCC patients in China [18–20]. Besides, the results if PIVKA-II performs better than AFP for HCC diagnosis and prognosis have been controversial up to now. Therefore, we enrolled a sufficient sample size of HCC patients as well as integrated controls including LC and BLD, to identify the performance of PIVKA-II, AFP, and their combination for HCC diagnosis.

In the present study, our results revealed that the diagnostic performance of PIVKA-II was better than AFP for Chinese HCC patients. In the whole HCC cohort, the sensitivity and AUC were 89.0% and 0.90 (95% CI 0.87-0.94) for PIVKA-II and 68.8% and 0.79 (95% CI 0.74-0.84) for AFP. In the early stage HCC patients, the sensitivity and AUC were 74.5% and 0.83 (95% CI 0.77-0.89) for PIVKA-II and 60.9% and 0.75 (95% CI 0.68-0.81) for AFP. When AFP and PIVKA-II were used in combination, the diagnostic accuracy significantly improved not only in whole HCC cohort but also in the early stage group. Moreover, in the AFP-negative HCC patients, PIVKA-II still showed excellent diagnostic performance with the AUC 0.81 (95% CI 0.72-0.89). Our results are similar to the previously published studies [9, 21, 22]; serum PIVKA-II was obviously superior to AFP for HCC screening and provides further evidence that “AFP + PIVKA-II” was an effective blood-based biomarker facilitating diagnoses of HCC.

An elevated PIVKA-II level was reported to function as a predictive factor for microvascular invasion [18, 23]. However, our data indicated that there was no relationship between PIVKA-II levels and microvascular invasion. More large clinical studies are needed to verify these results in the future. Interestingly, our current study found that serum PIVKA-II level was significantly correlated with an increased risk of PVTT (OR = 4.890, 95% CI 1.361-38.169; *P* = 0.020), which may help to predict the prognosis of patients with HCC and guide therapeutic strategy.

Compared with previous research, this study offers several strengths. First, many previous studies focused on HCC patients in Japan as well as Western countries [15, 16, 19], and the diagnostic accuracy of PIVKA-II was debatable due to different demography and etiology of liver diseases. There, we enrolled a total of 428 Chinese participants including HCC patients, LC, and BLD cases, and the sample size is more sufficient to elucidate the diagnostic role of PIVKA-II and AFP for Chinese HCC patients. Second, we fully and comprehensively validated serum PIVKA-II and AFP levels as diagnostic biomarkers in three groups, including whole HCC cohort, the early stage group, and AFP-negative HCC patients. Third, recently published studies rarely reported the relationship between serum PIVKA-II expression level and portal vein tumor thrombus. Our data revealed that elevated PIVKA-II serum level could independently predict an increased risk of HCC accompanied by PVTT.

However, there are some limitations in our study. First, our study was designed retrospectively. More prospective clinical trials are required to validate the results. Second, the underlying biological mechanism of PIVKA-II in HCC and the reasons why increased PIVKA-II expression level is correlated with PVTT have not yet been elucidated. Third, we did not perform further follow-up to determine the association between dynamic changes of PIVKA-II and HCC prognostic value.

## 5. Conclusion

The current study confirmed that PIVKA-II is an excellent diagnostic biomarker for Chinese HCC patients and could be used as a predictor for HCC accompanied by PVTT. Notably, “AFP + PIVKA-II” achieved high specificity and sensitivity for detecting HCC, particularly in the detection of early stage HCC. Based on the evidences of the current study, we therefore strongly recommend that PIVKA-II should be routinely tested for managing Chinese HCC patients.

## Figures and Tables

**Table 1:** Baseline characteristics of all the studied groups.

	HCC (*n* = 308)	LC (*n* = 60)	BLD (*n* = 60)	*P* value
Age (years)	51.4 ± 12.2	46.6 ± 10.3	42.53 ± 12.3	*P* < 0.001
Gender (male/female)	271/37	48/12	46/14	*P* = 0.014
Total bilirubin (mg/dl)	16.64 ± 9.81	10.87 ± 3.85	7.96 ± 2.31	*P* = 0.026
AFP (ng/ml)	235.30 (0.70-121000.00)	13.16 (1.46-56.50)	4.99 (0.60-45.78)	*P* < 0.001
PIVKA-II (mAU/ml)	1805.50 (9.00-75000.00)	24.00 (11.00-45.00)	25.00 (12.00-46.00)	*P* < 0.001
AST (U/L)	49.70 (11.80-938.90)	55.60 (11.00-298.00)	18.80 (12.50-39.6)	*P* = 0.034
ALT (U/L)	40.40 (8.6-1265.7)	38.10 (4.00-158.50)	15.85 (3.20-45.30)	*P* = 0.029
ALB (g/L)	39.20 (27.70-52.50)	40.55 (23.90-50.20)	51.20 (33.40-69.20)	*P* = 0.076
PT-INR	1.47 (0.75-1.62)	1.55 (0.75-1.94)	1.89 (0.95-2.44)	*P* = 0.130

Data are the mean ± standard deviation or median (minimum-maximum) values. HCC: hepatocellular carcinoma; LC: liver cirrhosis; BLD: benign liver disease; AFP: alpha-fetoprotein; PIVKA-II: prothrombin induced by vitamin K absence II; AST: aspartate aminotransferase; ALT: alanine aminotransferase; ALB: albumin; PT-INR: prothrombin time-international normalized ratio.

**Table 2:** Diagnostic accuracy of the AFP and PIVKA-II in HCC.

	Se (%)	Sp (%)	PPV (%)	NPV (%)	Accuracy (%)	AUC (95% CI)	Kappa
*HCC vs. all controls*							
AFP	68.8	87.6	94.2	52.7	74.5	0.79 (0.74-0.84)	0.48
PIVKA-II	89.0	91.7	96.5	76.4	89.7	0.90 (0.87-0.94)	0.76
AFP + PIVKA-II	95.1	83.3	93.6	87.0	91.9	0.89 (0.85-0.93)	0.80
*HCC vs. LC*							
AFP	68.8	83.3	95.5	34.2	71.5	0.76 (0.70-0.82)	0.33
PIVKA-II	89.0	90.0	97.9	61.4	89.2	0.89 (0.85-0.94)	0.67
AFP + PIVKA-II	95.1	76.7	95.4	75.4	96.2	0.85 (0.78-0.92)	0.71

Se: sensitivity; Sp: specificity; PPV: positive predictive value; NPV: negative predictive value; AUC: area under the curve; CI: confidence interval; AFP: alpha-fetoprotein; PIVKA-II: prothrombin induced by vitamin K absence II; LC: HBV-related liver cirrhosis.

**Table 3:** Diagnostic accuracy of the AFP and PIVKA-II in early stage HCC.

	Se (%)	Sp (%)	PPV (%)	NPV (%)	Accuracy (%)	AUC (95% CI)	Kappa
*HCC vs. all controls*							
AFP	60.9	89.2	83.8	71.3	75.6	0.75 (0.68-0.81)	0.50
PIVKA-II	74.5	91.7	89.1	79.7	89.8	0.83 (0.77-0.89)	0.67
AFP + PIVKA-II	83.3	89.1	89.3	83.1	86.1	0.86 (0.81-0.91)	0.72
*HCC vs. LC*							
AFP	60.9	83.3	87.0	53.8	68.8	0.72 (0.64-0.80)	0.39
PIVKA-II	74.5	90.0	93.2	65.9	80.0	0.82 (0.75-0.88)	0.60
AFP + PIVKA-II	83.3	76.7	87.7	69.7	81.2	0.82 (0.74-0.89)	0.59

Se: sensitivity; Sp: specificity; PPV: positive predictive value; NPV: negative predictive value; AUC: area under the curve; CI: confidence interval; AFP: alpha-fetoprotein; PIVKA-II: prothrombin induced by vitamin K absence II; LC: HBV-related liver cirrhosis.

**Table 4:** Correlation between serum PIVKA-II level and clinical characteristics of HCC.

Characteristic	*N*	PIVKA-II ≤ 40 (mAU/ml)	PIVKA-II > 40 (mAU/ml)	*P* value
Age (years)				0.002^∗^
≤50	136	5	131	
>50	172	25	147	
Sex				0.056
Male	271	22	249	
Female	37	8	29	
Tumor size (cm)				<0.001^∗^
≤3	70	24	46	
3~5	43	2	41	
5~10	92	4	88	
>10	103	0	103	
Tumor number				0.010^∗^
Single	206	28	178	
Multiple	102	2	100	
Lymphatic metastasis				< 0.001^∗^
No	285	30	225	
Yes	23	0	23	
Distant metastasis				0.377
No	293	30	263	
Yes	15	0	15	
BCLC stage				<0.001^∗^
0	26	9	17	
A	84	17	67	
B	164	4	160	
C	34	0	34	
Tumor differentiation				0.027^∗^
Well-differentiated	22	9	13	
Moderately/poorly differentiated	158	26	94	
Microvascular invasion				0.576
No	120	9	111	
Yes	60	6	54	
PVTT				0.010^∗^
No	243	30	213	
Yes	65	0	65	

N: number of patients; AFP: alpha-fetoprotein; PIVKA-II: prothrombin induced by vitamin K absence II; PVTT: portal vein tumor thrombus; ^∗^significant difference (*P* < 0.05).

**Table 5:** Univariate analysis and multivariate analysis for PIVKA-II.

Parameters	Univariate analysis		Multivariate analysis	
	OR (95% CI)	*P*	OR (95% CI)	*P*
Age (≤ 50 vs. >50)	0.351 (0.153-0.803)	0.001	0.323 (0.120-0.864)	0.024^∗^
Sex (male vs. female)	0.975 (0.221-4.303)	0.930		
TBIL	1.025 (0.280-3.753)	0.971		
Tumor size	7.070 (3.246-21.101)	< 0.001	4.280 (1.952-9.384)	0.012^∗^
Tumor differentiation (well vs. moderately/poorly)	0.521 (0.135-2.013)	0.344		
Tumor multiplicity (single vs. multiple)	5.854 (1.742-19.621)	0.004	1.209 (0.287-5.092)	0.796
BCLC staging	2.890 (1.126-7.419)	0.020	1.319 (0.468-3.717)	0.600
AFP	2.156 (1.047-4.437)	0.037	2.062 (0.874-4.487)	0.069
PVTT	7.346 (2.579-20.921)	< 0.001	4.890 (1.361-38.169)	0.020^∗^

PIVKA-II: prothrombin induced by vitamin K absence II; TBIL: total bilirubin; AFP: alpha-fetoprotein; CI: confidence interval; PVTT: portal vein tumor thrombus; ^∗^significant difference (*P* < 0.05).

**Table 6:** Predictive factors of PVTT in hepatocellular carcinomas.

Parameters	Univariate analysis		Multivariate analysis	
	OR (95% CI)	*P*	OR (95% CI)	*P*
Age (≤ 50 vs. > 50)	0.443 (0.253-0.774)	0.040	0.414 (0.214-0.799)	0.009^∗^
Sex (male vs. female)	1.036 (0.449-2.388)	0.934		
TBIL	1.032 (1.006-1.058)	0.016	1.025 (0.995-1.056)	0.100
Tumor size	3.845 (1.370-10.789)	0.011	2.515 (0.258-24.55)	0.428
Tumor differentiation (well vs. moderately/poorly)	1.025 (0.280-3.753)	0.971		
Tumor multiplicity (single vs. multiple)	3.065 (1.745-5.381)	< 0.001	2.118 (1.097-4.089)	0.025^∗^
BCLC staging	6.053 (1.522-24.071)	0.011	0.211 (0.012-3.824)	0.292
AFP	2.641 (1.312-5.318)	0.007	1.536 (0.710-3.323)	0.276
PIVKA-II	7.346 (2.579-20.921)	< 0.001	4.890 (1.361-38.169)	0.020^∗^

TBIL: total bilirubin; AFP: alpha-fetoprotein; PIVKA-II: protein induced by vitamin K absence or antagonist-II; CI: confidence interval; PVTT: portal vein tumor thrombus; ^∗^Significant difference (*P* < 0.05).

## Data Availability

The data used to support the findings of this study are available from the corresponding author upon reasonable request.

## References

[1] Bray F., Ferlay J., Soerjomataram I., Siegel R. L., Torre L. A., Jemal A. (2018). Global Cancer Statistics 2018: GLOBOCAN Estimates of Incidence and Mortality Worldwide for 36 Cancers in 185 Countries. *CA: A Cancer Journal for Clinicians*.

[2] Bruix J., Sherman M. (2011). Management of hepatocellular carcinoma: an update. *Hepatology*.

[3] Harding J. J., Abou-Alfa G. K. (2015). Treating advanced hepatocellular carcinoma: how to get out of first gear. *Cancer*.

[4] Giannini E. G., Marenco S., Borgonovo G. (2012). Alpha-fetoprotein has no prognostic role in small hepatocellular carcinoma identified during surveillance in compensated cirrhosis. *Hepatology*.

[5] Colli A., Fraquelli M., Casazza G. (2006). Accuracy of ultrasonography, spiral CT, magnetic resonance, and alpha-fetoprotein in diagnosing hepatocellular carcinoma: a systematic review. CME. *American Journal of Gastroenterology*.

[6] Kasahara A., Hayashi N., Fusamoto H. (1993). Clinical evaluation of plasma des-gamma-carboxy prothrombin as a marker protein of hepatocellular carcinoma in patients with tumors of various sizes. *Digestive Diseases and Sciences*.

[7] Ji J., Wang H., Li Y. (2016). Diagnostic evaluation of des-gamma-carboxy prothrombin versus *α*-fetoprotein for hepatitis B virus-related hepatocellular carcinoma in China: a large-scale, multicentre study. *PLoS One*.

[8] Park H., Park J. Y. (2013). Clinical significance of AFP and PIVKA-II responses for monitoring treatment outcomes and predicting prognosis in patients with hepatocellular carcinoma. *BioMed Research International*.

[9] Lim T. S., Kim D. Y., Han K. H. (2016). Combined use of AFP, PIVKA-II, and AFP-L3 as tumor markers enhances diagnostic accuracy for hepatocellular carcinoma in cirrhotic patients. *Scandinavian Journal of Gastroenterology*.

[10] Park S. J., Jang J. Y., Jeong S. W. (2017). Usefulness of AFP, AFP-L3, and PIVKA-II, and their combinations in diagnosing hepatocellular carcinoma. *Medicine*.

[11] Choi J. Y., Jung S. W., Kim H. Y. (2013). Diagnostic value of AFP-L3 and PIVKA-II in hepatocellular carcinoma according to total-AFP. *World Journal of Gastroenterology*.

[12] Chan S. L., Chong C. C., Chan A. W. (2016). Management of hepatocellular carcinoma with portal vein tumor thrombosis: review and update at 2016. *World Journal of Gastroenterology*.

[13] Cheng S., Yang J., Shen F. (2016). Multidisciplinary management of hepatocellular carcinoma with portal vein tumor thrombus - Eastern Hepatobiliary Surgical Hospital consensus statement. *Oncotarget*.

[14] Zhang Z. M., Lai E. C., Zhang C. (2015). The strategies for treating primary hepatocellular carcinoma with portal vein tumor thrombus. *International Journal of Surgery*.

[15] Zakhary N. I., Khodeer S. M., Shafik H. E., Abdel Malak C. A. (2013). Impact of PIVKA-II in diagnosis of hepatocellular carcinoma. *Journal of Advanced Research*.

[16] Ricco G., Cavallone D., Cosma C. (2018). Impact of etiology of chronic liver disease on hepatocellular carcinoma biomarkers. *Cancer Biomarkers*.

[17] Xu F., Zhang L., Xu Y. (2020). Hypermethylation of SCAND3 and Myo1g gene are potential diagnostic biomarkers for hepatocellular carcinoma. *Cancers*.

[18] Poté N., Cauchy F., Albuquerque M. (2015). Performance of PIVKA-II for early hepatocellular carcinoma diagnosis and prediction of microvascular invasion. *Journal of Hepatology*.

[19] Caviglia G. P., Ribaldone D. G., Abate M. L. (2018). Performance of protein induced by vitamin K absence or antagonist-II assessed by chemiluminescence enzyme immunoassay for hepatocellular carcinoma detection: a meta-analysis. *Scandinavian Journal of Gastroenterology*.

[20] Wang X., Zhang W., Liu Y. (2017). Diagnostic value of prothrombin induced by the absence of vitamin K or antagonist-II (PIVKA-II) for early stage HBV related hepatocellular carcinoma. *Infectious Agents and Cancer*.

[21] Wu J., Xiang Z., Bai L. (2018). Diagnostic value of serum PIVKA-II levels for BCLC early hepatocellular carcinoma and correlation with HBV DNA. *Cancer Biomarkers*.

[22] Ma X. L., Zhu J., Wu J. (2018). Significance of PIVKA-II levels for predicting microvascular invasion and tumor cell proliferation in Chinese patients with hepatitis B virus-associated hepatocellular carcinoma. *Oncology Letters*.

[23] Masuda T., Beppu T., Okabe H. (2016). Predictive factors of pathological vascular invasion in hepatocellular carcinoma within 3 cm and three nodules without radiological vascular invasion. *Hepatology Research*.

